# Estimating and Explaining the Prevalence of Tuberculosis for Asylum Seekers Upon Their Arrival in Germany

**DOI:** 10.1007/s10903-020-01134-y

**Published:** 2020-12-23

**Authors:** S. Stadtmüller, J. Schröder, S. Ehlers

**Affiliations:** 1 GESIS – Leibniz-Institute for the Social Sciences, P.O.Box 12 21 55, 68072 Mannheim, Germany; 2 Research Center Borstel, Leibniz Lung Center, Borstel, Germany

**Keywords:** Tuberculosis, Asylum seekers, Germany, At-entry prevalence, Healthy migrant effect

## Abstract

Up until recently incidences of tuberculosis (TB) had been declining for many years in Germany. The rise in TB cases coincided with a large increase in the number of people applying for asylum. We combine data from various sources to estimate the at-entry prevalence of TB for asylum seekers from 18 countries of origin and rely on survey data to explain the varying risk of suffering from TB. Our results reveal that asylum seekers from Eastern Africa show a much higher risk of suffering from TB than asylum seekers from Afghanistan, Syria, or Iraq. The survey data suggests that asylum seekers from Africa were by far more underprivileged in their respective countries of origin and experienced a higher risk of contracting TB on their way to Germany. Information about the socio-economic situation and the circumstances of the journey to Germany may help to improve TB surveillance.

## Introduction

**Fig. 1:**
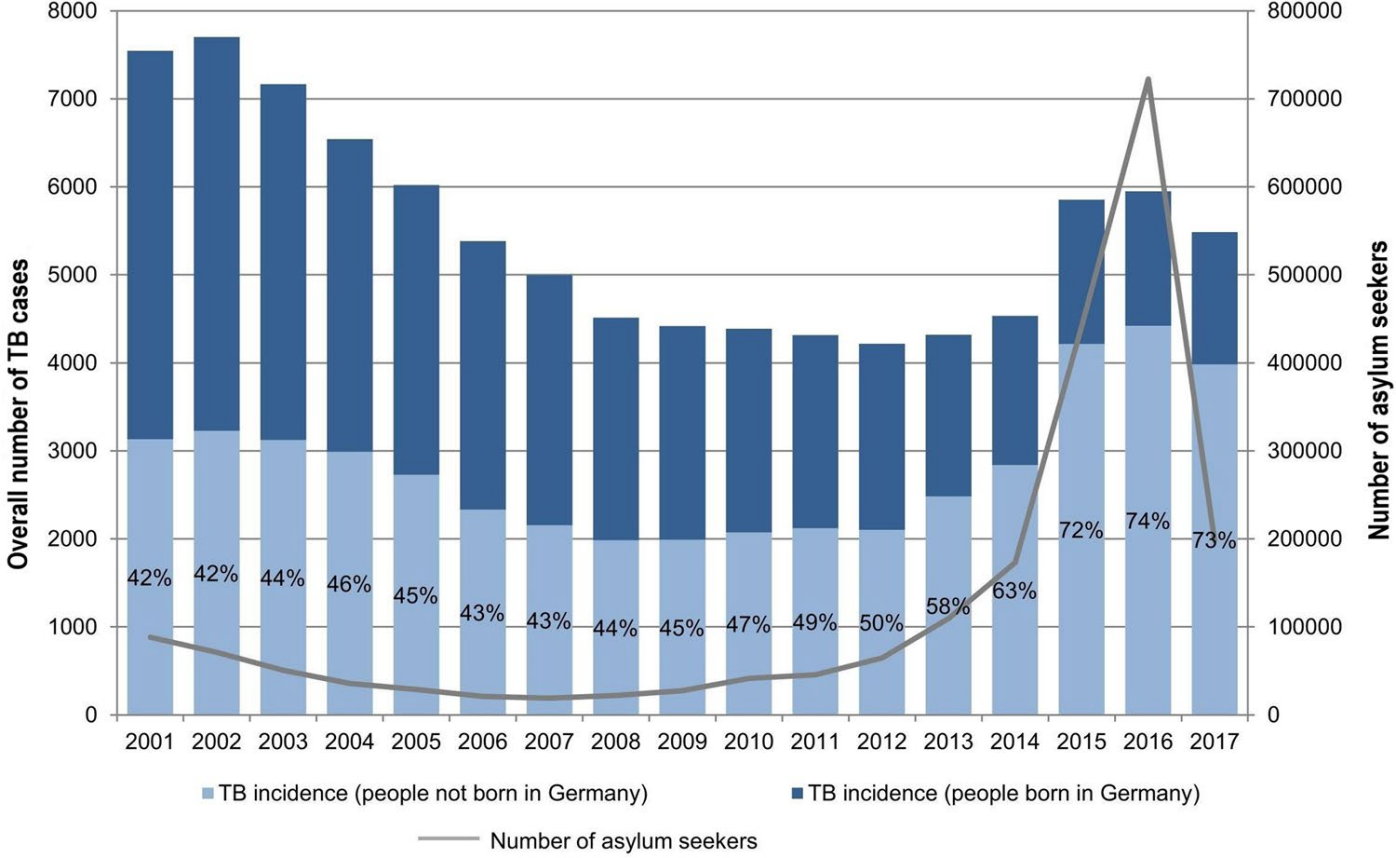
TB incidence in Germany by country of birth and applications for asylum, 2001–2017

For many years tuberculosis (TB) incidence rates in Germany had been sinking. While in 2002, 9.3 out of 100,000 people suffered from TB in Germany, the incidence rate dropped to 5.5 in 2008 and remained fairly constant until 2014. In 2015 and 2016, however, incidence rates rose remarkably up to 7.1 (in 2015) and 7.2 (in 2016) per 100,000 people, with almost 6000 cases of TB in each year (compared with 4529 in 2014) [1]. This increase coincided with a large increase in the number of people applying for asylum [2]. This number more than quadrupled between 2014 and 2016 with roughly 722,000 people applying for asylum in Germany for the first time in 2016 (in 2014: 173,000). Although the share of TB patients not born in Germany has consistently been high throughout the last decades, it also rose from roughly 60% in 2012 and 2013 to more than 70% between 2015 and 2017 [1] (see Fig. 1). These developments seem to suggest a connection between the increase in TB incidences and the rising number of people who immigrated to Germany.

In Germany, the Robert Koch Institute (RKI) is responsible for the surveillance of infectious diseases. Local public health offices must notify cases of active TB to the RKI and the mode of case finding (i.e. reason for diagnosis) is part of the notified information. One possible category for the mode of case finding is chest X-ray screening for people who enter shared accommodations. This screening procedure is prescribed by the German Law on the Prevention and Control of Infectious Diseases and is carried out for asylum seekers who normally move into shared accommodation after their arrival in Germany. In sum, 1262 cases of TB were detected by this means in 2015 among asylum seekers, a steep increase from 2013 with only 216 detected cases [3, 4]. However, the total number of individuals screened is not reported to the RKI by the local public health offices as this information is not notifiable.

In order to approach the relative risk of asylum seekers from different countries of origin to suffer from an active TB upon their arrival in Germany, our first objective is to estimate at-entry prevalence rates of TB for asylum seekers from 18 countries of origin. In a second step, we compare the estimated prevalence rates with the country-specific TB incidence rates made public through the WHO [5] and set out to explain the country differences in the relationship between these two figures. Since vulnerability to TB is known to be related with poverty, we first take into account asylum seekers’ socio-economic situation compared to other people living in their country of origin [6, 7]. According to the “healthy migrant effect”, migration is a highly selective process in which healthy people predominantly take on the burden of leaving their country [8–10]. However, this may hold true for asylum seekers from some countries but not from others and may therefore help to explain some of the country differences in the relationship between estimated prevalence rates and country-specific TB incidence rates. Moreover, we consider the risk of contracting TB while immigrating to Germany. This might prove particularly important given that asylum seekers on their way to Germany are exposed to adverse living conditions and to crowded housing situations, which presumably both increase their risk of contracting TB. If the estimated at-entry prevalence rates reflect varying circumstances of the journey for asylum seekers from different countries of origin one would expect TB prevalence rates to be higher for those asylum seekers with relatively long and burdensome journeys. In general, if we find evidence that both, the asylum seekers’ relative socio-economic situation in their home country as well as the migration process have an important influence on TB burden upon arrival in the host country, TB control and prevention could be enhanced by not solely relying on the published TB incidence rates of asylum seekers’ countries of origin but by taking into account one or both of these factors.

## Methods

To estimate the at-entry prevalence rates of TB we combine data provided by two German governmental institutions, namely the Robert Koch Institute (RKI) and the Federal Office for Migration and Refugees (BAMF). RKI’s annual reports on TB epidemiology in Germany publish the ten countries of origin with the largest absolute number of asylum seekers diagnosed with TB during the at-entry chest X-ray examination as well as the respective number of cases [1]. We limited our analysis to the timespan from 2014 to 2016 as the influx of refugees to Germany was most pronounced then. Since the ten countries of origin with most TB cases varied by year, we included all countries which were in this list in at least 1 year and asked the RKI for the respective value(s) for the outstanding year(s). Due to data protection issues, we only received the cumulative numbers of TB cases over the time span 2014–2016 for these countries. In sum, we gathered data for 18 countries of origin.^1^ BAMF provided us with the number of first time asylum applications for these countries during the same time span. This data is used to approximate the number of asylum seekers by country of origin who received a chest X-ray examination on their arrival in Germany.^2^ Based on these numbers and the country specific numbers of TB cases provided by the RKI, we estimate the at-entry TB prevalence rates.

For the country-specific TB incidence rates we draw on the WHO TB burden estimates [11]. We use the incidence for 2015, i.e., the midterm of our timespan of interest for the comparison with the estimated prevalence rates.

To explain the country differences in the relationship between at-entry TB-prevalence rates of asylum seekers and the TB incidence rates in their country of origin, we rely on data from the IAB-BAMF-SOEP Refugee Survey [12, 13]. In 2016, more than 4000 randomly selected adults (aged 17 and above) who had recently migrated to Germany were surveyed by specifically trained interviewers. Since the survey aimed at reflecting the structure of asylum seekers to Germany by country of origin, only countries with a high influx of asylum seekers to Germany are represented in the survey by a substantial number of respondents. These countries include Syria (n = 2229), Iraq (n = 594), Afghanistan (n = 573), and Eritrea (n = 243). One survey question asks respondents to assess their own economic situation in their country of origin compared to other people living there. Moreover, we rely on the questions on how many days it took respondents to travel to Germany and in which countries they stopped on their way to Germany for more than three months.

For our study, no ethical approval is required since we use (1) aggregated official data that is publicly available and (2) survey data that was collected by third parties in accordance with the data protection regulations in Germany.

## Results

For the 18 countries under study, Table 1 displays the cumulative number of TB cases detected via chest X-ray screening and the number of asylum seekers who came to Germany between 2014 and 2016. These numbers are used to calculate the at-entry prevalence rates of TB, displayed in the fourth column. The greatest risk of having an active TB when entering Germany have asylum seekers from Somalia with a prevalence rate of 2968 (per 100,000 asylum seekers) followed by Ethiopia (2090), Senegal (1322) and Gambia (1219).

**Table 1:** Estimated at-entry prevalence rates of asylum seekers compared with the country-specific TB incidences

Country	Number of at-entry TB cases 2014–2016	Number of asylum applications 2014–2016	At-entry prevalence rate	TB-incidence (WHO) and CI		Ratio and CI.	
Afghanistan	294	167,509	176	189	[122–270]	0.9	[0.7–1.4]
Albania	44	76,523	57	16	[14–19]	3.6	[3.0–4.1]
Ethiopia	91	4355	2090	192	[142–250]	10.9	[8.4–14.7]
Bosnia and Herzegovina	16	12,253	131	39	[30–49]	3.4	[2.7–4.4]
Eritrea	306	42,928	713	70	[32–123]	10.2	[5.8–22.3]
Gambia	87	7136	1219	174	[131–223]	7.0	[5.5–9.3]
Georgia	66	9143	722	99	[80–120]	7.3	[6.0–9.0]
Iraq	72	131,245	55	43	[38–49]	1.3	[1.1–1.4]
Iran	13	35,014	37	16	[12–20]	2.3	[1.9–3.1]
Morocco	36	7166	502	101	[87–117]	5.0	[4.3–5.8]
Macedonia	27	19,532	138	17	[13–21]	8.1	[6.6–10.6]
Nigeria	37	21,840	169	219	[143–311]	0.8	[0.5–1.2]
Pakistan	148	26,651	555	270	[180–378]	2.1	[1.5–3.1]
Russian Federation	66	20,653	320	67	[43–96]	4.8	[3.3–7.4]
Senegal	35	2647	1322	130	[92–174]	10.2	[7.6–14.4]
Serbia	65	40,271	161	21	[18–25]	7.7	[6.4–8.9]
Somalia	410	13,813	2968	274	[177–391]	10.8	[7.6–16.8]
Syria	280	464,239	60	19	[15–25]	3.2	[2.4–4.0]

The next two columns show the TB incidence rates published by WHO and their confidence intervals. It is evident that the countries of origin with high incidence rates vary substantially in their respective at-entry prevalence rates. For example, Afghanistan and Ethiopia have almost the same TB incidence rates (with values of 189 and 192, respectively). However, the at-entry prevalence rates are very different for the two countries with a value of 176 for asylum seekers from Afghanistan but 2090 for Ethiopians.

In order to systematically compare the estimated at-entry prevalence rate and the published incidence rate in the country of origin, we calculated the ratio of these two values for all countries. Of course, we are fully aware that prevalence and incidence rates measure similar yet different things. However, the calculation of the ratio helps to get a first impression on how TB burden in the asylum seekers’ home countries (as reflected by the incidence rates) differs from the TB burden of asylum seekers from the respective countries. In sum, for 16 of 18 researched countries, this ratio is above 1 indicating that the estimated at-entry prevalence of TB for asylum seekers from those countries is higher than the published TB incidence rate in the respective country of origin. In four of the researched countries this ratio even exceeds the factor ten, meaning that the prevalence rate is more than ten times higher than the respective incidence rates. Interestingly, all of those four countries belong to Africa with three from the eastern (Ethiopia, Eritrea and Somalia) and one from the western part (Senegal). Markedly higher TB prevalence rates can also be made out for asylum seekers from Gambia and Morocco, from South Eastern Europe (Serbia, Macedonia) and from Eastern Europe (Russia, Georgia). In contrast, the situation for asylum seekers from Southern Asia (Afghanistan and Pakistan) and from the Middle East (Syria, Iraq, and Iran) is such that the estimated prevalence rates are not more than three times higher than the respective reported incidence rates.

**Fig. 2:**
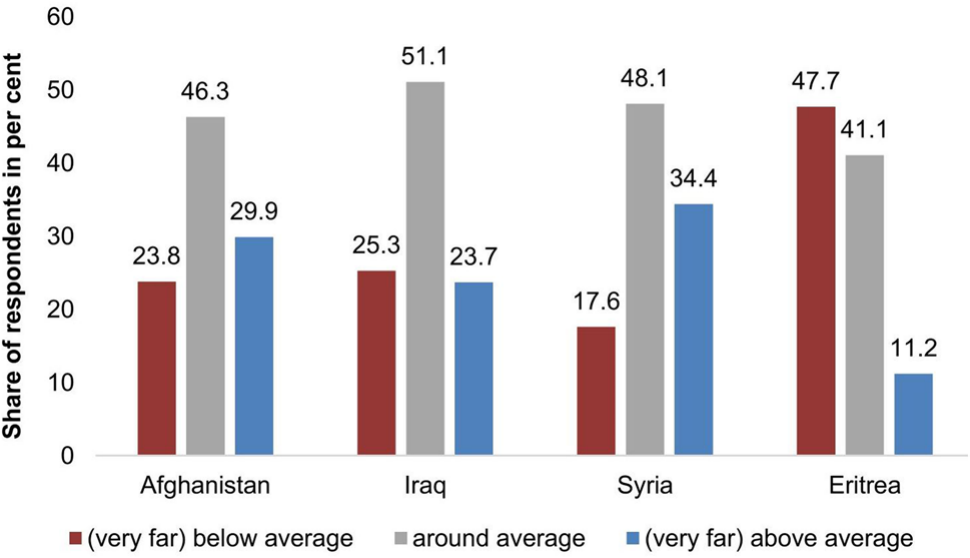
Asylum seekers’ self-assessment of their socio-economic situation compared to other people living in their country of origin

In the following we investigate whether the asylum seekers’ relative socio-economic situation in their home country and country differences in the migration process can contribute to the explanation of the high variability of our calculated ratios *between* the 18 countries. More specifically, we are interested in whether these factors could account for the high ratios between at-entry prevalence rate and country of origin’s incidence rate for eastern African countries as compared to the low ratios for countries like Afghanistan, Iraq, or Syria. The IAB-BAMF-SOEP Refugee Survey provides us with data for one of the eastern African countries, namely Eritrea, and for the three latter countries. Figure 2 indicates that among Eritrean asylum seekers a large proportion was underprivileged in their home country: 48% responded that they were worse off than other people in their country of origin while only 11% responded they were better off. This is in contrast to Afghanistan, Iraq, or Syria. For these countries asylum seekers’ relative socio-economic status shows a balanced or even slightly positive picture with only between 18% (Syria) and 25% (Iraq) reporting that they were worse off than other people in their home country.

**Fig. 3:**
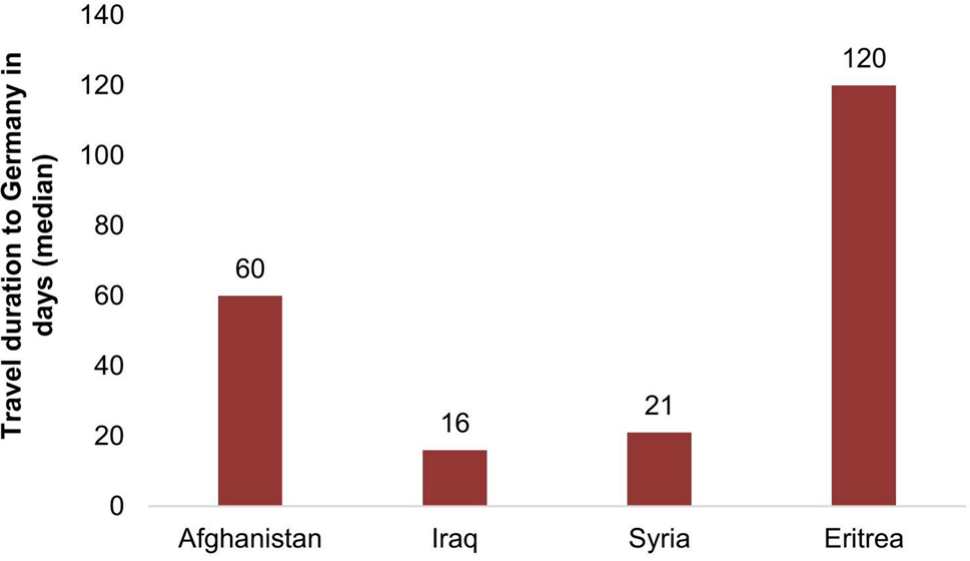
Duration of the flight to Germany for asylum seekers from different countries of origin

When it comes to the question how many days it took asylum seekers to get to Germany from their country of origin we can, again, observe large differences (Fig. 3): Asylum seekers from Eritrea showed by far the highest value, with a median of 120 days as compared to between 16 (Iraq) and 60 (Afghanistan) days in the other countries. Respondents were also asked in which countries they stopped on their way to Germany for at least 3 months. Almost three quarters of the surveyed asylum seekers from Eritrea had stopped at least in one country for more than 3 months. In all other groups this proportion was much smaller. At the same time, 29% of the asylum seekers from Eritrea stopped at least for 3 months in Libya, where living conditions are known to be extremely unfavorable [14].^3^ In contrast, asylum seekers from the other three countries mostly took the route via Turkey, a country in which the living conditions are presumably far more favorable than in Libya.

## Discussion

With regard to our estimated at-entry prevalence rates, we first observe them to be higher than the published country-specific incidence rates in 16 out of our 18 studied countries of origin. This is hardly surprising since we would expect a high TB burden in a population with restricted access to health care provision either in their country of origin (e.g., due to war or persecution) or during their journey—like it is the case for our population of interest. Moreover, we found that prevalence rates are many times higher than the home countries’ incidence rates for asylum seekers from eastern Africa, but not for asylum seekers from the Middle East and Southern Asia. These findings are in line with three regional studies carried out in Germany [15–17] and with data gathered in other host or transit countries (cp. [18]).

Apart from comparing at-entry prevalence rates for asylum seekers from 18 countries of origin with the published incidence rates in their home country, we also tried to explain why the prevalence rates differ considerably from the incidence rates for some countries but not for others. Data from a German Refugee Survey revealed that asylum seekers from eastern Africa were by far more underprivileged in their respective countries of origin than was the case for asylum seekers from Afghanistan, Syria, or Iraq. Since health status in general, and proneness to TB in particular are strongly related to poverty, this may help to explain why prevalence rates for asylum seekers from African countries are that high, contradicting the “healthy migrant” hypothesis.

Moreover, the survey data indicates that asylum seekers from Eritrea on the one hand and those from Syria, Iraq, and Afghanistan on the other vary with regard to their risk of contracting TB on their way to Germany. Eritrean asylum seekers needed much longer for their way to Germany than refugees from Syria, Afghanistan and from the Iraq and mostly took the route via Libya.^4^ Adding to the duration and route of their journeys are certainly the crowded accommodations they were in for a very long time. Adverse housing situations as encountered during the journey presumably increase the risk of contracting TB (for a highly instructive example of transmission in a Libyan refugee camp, see [20]).^5^ Apart from that, other factors may also contribute to the much higher TB at-entry prevalence rate for asylum seekers from African countries. These include their presumably higher seroprevalence of HIV, making them more susceptible for TB [21]. Moreover, WHO data suggest that the share of extrapulmonary TB is considerably lower in African countries than in the Middle East (e.g., 48% in Iraq, 45% in Syria, and 35% in Eritrea, 14% in Senegal, and 26% in Somalia [22]). Since our analyses focuses on pulmonary TB this may partly explain the between-country variation in the ratio between at-entry prevalence and TB incidence.

What do our results suggest for TB control and prevention in Germany? If we take our estimated prevalence rates at face value, the German practice of ruling out an active TB only once, i.e., when entering the country, may not be sufficient. This holds especially true for asylum seekers from most African countries. For these, yearly follow-up examinations are warranted because chest X-ray examinations are not able to exclude a latent infection with TB. Therefore, its result tells us nothing about the risk of the screened person to develop an active TB at a later time. In line with this, the literature suggests that there is a long-term impact of migration on the TB burden in host countries [23, 24]. Finally, a better data base for TB surveillance in Germany is necessary. An important step in this direction would be to routinely document the number of X-ray examinations carried out, disaggregated by (at least) country of origin, age, and gender. Additionally, information about the socio-economic situation and information about the duration of their journey and the countries in which they stopped on their way to Germany may also help to improve TB surveillance—not only in Germany but also in all other host countries.

## New Contributions to the Literature

In our study, we estimated the risk of suffering from TB for asylum seekers from 18 countries of origin upon their arrival in Germany. Although there are some regional studies sharing this objective, our contribution has the advantage of including all TB cases of asylum seekers detected during the chest X-ray examination from 2014 to 2016. Hence, it rests on a much broader information basis and thus minimizes potential errors. Moreover, we try to explain why asylum seekers from eastern African countries are particularly prone to suffering from TB when they arrive in Germany. For this, we make use of survey data that specifically asks about asylum seekers’ socio-economic situation compared with other people living in their home country and about the circumstances of their journey. To our knowledge, relating the risk of suffering from TB to these factors for asylum seekers from different regions of the world based on broad, empirical evidence is a new and promising approach that might help to increase preparedness and thus improve TB control and prevention in times of high migration.

## Footnotes

1 Our method of country selection implies that we relied on TB burden rather than on the influx of migrants for selecting the countries we studied. Therefore, countries with a high influx of migrants but with a negligible TB burden are systematically underrepresented in our analysis. However, the 18 countries account for 81% (2014), 88% (2015) and 86% (2016) of all asylum applications in the respective years, thus covering the vast majority of migration inflow to Germany in the period of interest.

2 As Fig. 1 suggests most people applied for asylum in 2016. However, the influx of refugees to Germany was strongest in 2015. Thus the numbers are somewhat distorted, which can be explained by the circumstance that the BAMF was not able to record all applications in 2015. Rather, many people who had already come to Germany in 2015 were recorded as asylum seekers only in 2016. Hence, there might be a considerable time gap between the X-ray examination (which is supposed to be carried out “at-entry”) and the refugees’ application for asylum. Therefore, it is reasonable to pool the data for our three years of interest and to estimate prevalence rates that capture the entire timespan 2014–2016 in order to avoid distorting results for each individual year.

3 Presumably, the share of people from Eritrea (or from eastern Africa in general) who stopped in Libya on their way to Germany is substantially higher. In the survey, however, only countries in which asylum seekers stopped for at least 3 months were counted as transit countries.

4 The survey data also suggests that the duration of the transit-country stop correlates with financial resources. One possible explanation is that asylum seekers with fewer resources need to make money in the transit country to finance the crossing and thus have to stay there for a longer period of time [19].

5 While this explanation cannot be applied to people from Macedonia, Serbia, or Georgia we believe that for people from those countries, their relative socio-economic situation (compared with other people living in their country of origin) plays an important role in explaining their high at-entry prevalence rate. Although only few respondents from these countries participated in the survey on asylum seekers, the results indicate that people from South Eastern Europe and from Eastern Europe are extremely underprivileged. Furthermore, in some qualitative interviews we carried out with health care providers in Germany, we received some indications that travelling to Germany for seeking health care is more common for people from those than for people from other regions. This may also contribute to the higher prevalence rates.
